# The genetic mechanisms underlying the convergent evolution of pollination syndromes in the Neotropical radiation of *Costus* L.

**DOI:** 10.3389/fpls.2022.874322

**Published:** 2022-09-08

**Authors:** Eugenio Valderrama, Jacob B. Landis, Dave Skinner, Paul J. M. Maas, Hiltje Maas-van de Kramer, Thiago André, Nikolaus Grunder, Chodon Sass, Maria Pinilla-Vargas, Clarice J. Guan, Heather R. Phillips, Ana Maria Rocha de Almeida, Chelsea D. Specht

**Affiliations:** ^1^School of Integrative Plant Science, Section of Plant Biology and the L.H. Bailey Hortorium, Cornell University, Ithaca, NY, United States; ^2^BTI Computational Biology Center, Boyce Thompson Institute, Ithaca, NY, United States; ^3^Le Jardin Ombragé, Tallahassee, FL, United States; ^4^Section Botany, Naturalis Biodiversity Center, Leiden, Netherlands; ^5^Departamento de Botânica, Instituto de Ciências Biológicas, Universidade de Brasília, Brasília, DF, Brazil; ^6^Department of Biological Sciences, California State University, East Bay, Hayward, CA, United States; ^7^University and Jepson Herbaria, University of California, Berkeley, Berkeley, CA, United States

**Keywords:** correlated evolution, Costaceae, signatures of selection, Nanopore, targeted enrichment, Zingiberales

## Abstract

Selection together with variation in floral traits can act to mold floral form, often driven by a plant’s predominant or most effective pollinators. To investigate the evolution of traits associated with pollination, we developed a phylogenetic framework for evaluating tempo and mode of pollination shifts across the genus *Costus* L., known for its evolutionary toggle between traits related to bee and bird pollination. Using a target enrichment approach, we obtained 957 loci for 171 accessions to expand the phylogenetic sampling of Neotropical *Costus*. In addition, we performed whole genome resequencing for a subset of 20 closely related species with contrasting pollination syndromes. For each of these 20 genomes, a high-quality assembled transcriptome was used as reference for consensus calling of candidate loci hypothesized to be associated with pollination-related traits of interest. To test for the role these candidate genes may play in evolutionary shifts in pollinators, signatures of selection were estimated as *dN/dS* across the identified candidate loci. We obtained a well-resolved phylogeny for Neotropical *Costus* despite conflict among gene trees that provide evidence of incomplete lineage sorting and/or reticulation. The overall topology and the network of genome-wide single nucleotide polymorphisms (SNPs) indicate that multiple shifts in pollination strategy have occurred across *Costus*, while also suggesting the presence of previously undetected signatures of hybridization between distantly related taxa. Traits related to pollination syndromes are strongly correlated and have been gained and lost in concert several times throughout the evolution of the genus. The presence of bract appendages is correlated with two traits associated with defenses against herbivory. Although labellum shape is strongly correlated with overall pollination syndrome, we found no significant impact of labellum shape on diversification rates. Evidence suggests an interplay of pollination success with other selective pressures shaping the evolution of the *Costus* inflorescence. Although most of the loci used for phylogenetic inference appear to be under purifying selection, many candidate genes associated with functional traits show evidence of being under positive selection. Together these results indicate an interplay of phylogenetic history with adaptive evolution leading to the diversification of pollination-associated traits in Neotropical *Costus*.

## Introduction

Recognition of the important role that pollinator interactions play in the diversification of flowering plants traces back to observations by Darwin (1892). Selection can act to mold the characteristics of flowers driven by their predominant or most effective pollinators (Stebbins, 1970), while floral antagonists and abiotic factors may also drive selection in parallel or opposing directions (Van der Niet et al., 2014; Caruso et al., 2019; Dellinger, 2020). The combination of traits (e.g., morphology, color, scent, size, and rewards) associated with particular pollinator groups are often referred to as pollination syndromes (Faegri and Pijl, 1979; Fenster et al., 2004; Rosas-Guerrero et al., 2014). Although the validity of the concept of pollination syndromes has been debated and some studies have not been able to predict pollinators using floral traits either alone or in combination, meta-analyses confirm that there is a higher predictability of pollination syndromes in plants that are distributed in the tropics and associated with bats, bees, or hummingbirds (Rosas-Guerrero et al., 2014; Ashworth et al., 2015). The possibility of both specialized pollination and of frequent shifts in pollination syndromes has been associated with increased opportunities for reproductive isolation (Serrano-Serrano et al., 2017; Fragoso-Martínez et al., 2018) and higher diversification rates (Lagomarsino et al., 2016; Xue et al., 2020). However, in some lineages there is not a clear association between phenotypic traits important for pollination or reproductive mode and rates of diversification (Landis et al., 2018; Moein et al., 2021).

A classic example of plants bearing traits associated with discrete pollinator guilds is the Neotropical section of the genus *Costus* L., a genus of tropical monocots comprising approximately 80 described species (Maas, 1972; Maas-van de Kamer et al., 2016). The genus is currently divided into a paraphyletic African grade and a large Neotropical clade, the latter of which radiated rapidly after a single long-distance dispersal event from Africa (Specht, 2006; André et al., 2016). Two main pollination syndromes have recursively evolved within the Neotropical *Costus* clade: “mellitophilous” (bee pollinated) species exhibiting flowers with a wide floral tube bordered by a broad labellum that is either yellow or white, typically marked by lateral coloration patterns and a central landing platform bearing a nectar guide or honey mark; and “ornithophilous” (bird pollinated) species, with narrow tubular flowers that are typically yellow, orange, pink, or red in color and lack any lateral markings (Maas, 1972; Specht et al., 2001). Mellitophilous flowers are most often subtended by green, often leafy, bracts while ornithophilous flowers are subtended by bright red, orange, or yellow bracts. Previous phylogenetic studies have estimated multiple shifts in pollination syndromes during the evolution of Neotropical *Costus*, including shifts to mellitophilous pollination syndromes and subsequent regains of ornithophilous flowers (Specht, 2006; Salzman et al., 2015; Valderrama et al., 2020; Vargas et al., 2020), mainly through sympatric speciation events (André et al., 2016). These results suggest that shifts in traits leading to distinct pollination syndromes, together with reproductive isolation *via* reinforcement, may account for the rapid radiation observed in this Neotropical clade.

In addition to ornithophilous species bearing traits that suggest adaptation to hummingbird pollination, there is evidence that some of the ornithophilous traits may also function in a process known as “bee avoidance” (Lunau et al., 2011). Bees are abundant visitors of flowers especially in tropical regions (Bawa, 1990; Renner and Feil, 1993), however they are often found to be less effective pollinators than hummingbirds (Castellanos et al., 2004; Freitas et al., 2006). The bee avoidance hypothesis suggests that certain traits that we associate with hummingbird-pollinated plants may have evolved to discourage visitation by nectar-robbing insects rather than to attract hummingbirds (Raven, 1972; Lunau and Maier, 1995). Rodríguez-Gironés and Santamaría (2004) showed that bees have limited ability to distinguish red-reflecting colors from the background, resulting in their inability to see red or darkly pigmented orange flowers. This results in their exclusion from red-reflecting flowers, creating a private channel of communication with birds and leaving avian pollinators free of competition for the nectar food source. The potential for bee avoidance behavior has been documented for *Costus*; in polymorphic populations of *Costus arabicus* L. bearing white and dark pink flowers, Bergamo et al. (2016) demonstrated that bees preferentially visited white flowers while hummingbirds show no preference for white or pink flowers. Given the number of evolutionary events in which bird pollination has evolved from bee pollination, Neotropical *Costus* provides an interesting case for further investigation into the evolutionary drivers of trait evolution, whether the traits actively attract effective “specialist” pollinators or prevent visitation by less effective generalists (Schemske and Horvitz, 1984; Thomson, 2003).

Another dimension of selective pressure on the floral morphology of Neotropical *Costus* comes in the form of anti-herbivory strategies. Many *Costus* species have evolved the ability to attract ants *via* the production of nectar by extrafloral glands located on the inflorescence bracts. The presence of ants is a well-documented strategy against herbivory in many plant species (e.g., Pringle et al., 2012), and has been shown to increase the viability of seeds and thereby the fitness of tropical plants by deterring seed herbivory (Rosumek et al., 2009). An example of this is seen in *Costus woodsonii* Maas, where ants deter flies (*Euxesta* sp.; Ulidiidae) from laying eggs on or within the bracts, thereby reducing larval predators that feed on the fruits and damage tissues which are critical for dispersal (arils) and reproduction (seeds) (Schemske, 1980, 1982). The presence of ants could also impact pollination, as bees tend to avoid flowers that are covered by ants in other Neotropical lineages (Nogueira et al., 2021). Similarly, flies are red-sensitive insects that have receptors with spectral sensitivity in those wavelengths (Lunau, 2014; Coimbra et al., 2020), suggesting the possibility that dark pigmentation traits associated with ornithophilous flowers could also impact interactions with egg-laying flies and larval predation. The evolution of *Costus* inflorescence morphology could thus reflect an interplay between selective pressures related to pollination biology and herbivory, influencing trait evolution and the tempo and mode of diversification across this tropical lineage.

Given the diversity of floral and inflorescence morphologies associated with key ecological and environmental interactions, *Costus* provides an outstanding model to study the genetic and genomic mechanisms underlying convergent evolution of organismal interactions, including pollination syndromes. Evolutionary developmental studies have suggested that floral developmental genes such as *AGAMOUS* (Almeida et al., 2015), *PISTILLATA* (Bartlett and Specht, 2010), and *YABBY*2/5 (Almeida et al., 2014; Morioka et al., 2015), as well as the gene regulatory networks in which they participate, may be involved in floral morphological and developmental shifts underlying the convergent evolution of pollination syndrome morphologies (Mendoza et al., 1999; Barrio et al., 2010). These genetic mechanisms may provide keys to understanding the processes that underlie the rapid radiation and diversification inherent to Neotropical *Costus*. Previous genome-level phylogenetic analyses of Neotropical *Costus* (Valderrama et al., 2020; Vargas et al., 2020) demonstrate challenges in recovering species-level resolution due to rapid radiation of morphological features defining species without concomitant divergence of molecular markers. By investigating the evolution and diversification of whole genomes and of candidate genes known to be involved in the evolution of morphological traits that are associated with species-level diversification of *Costus*, we may be able to detect selective pressures that drive the rapid radiation of this lineage and determine if similar mechanisms are responsible for the various transitions among observed morphological innovations. Examples in which trait diversity has been correlated with genomic signatures of selection to investigate mechanisms underlying trait evolution include local adaptation to coastal environments in the Australian Groundsel (*Senecio lautus* Willd.; Asteraceae; Roda et al., 2013) and adaptation to alpine environments in snapdragons (*Antirrhinum* L.; Plantaginaceae; Durán-Castillo et al., 2022).

Beyond looking at selection across individual genes, recent studies have shown evidence of genomic-level changes being driven explicitly by associations with pollinators; examples include higher genetic differentiation (F_*ST*_) in bee vs. vertebrate pollinated populations with increased geographic distance (Dellinger et al., 2022), and observed allele frequency changes following generations of bumble bee pollinated treatments when compared with the control (Frachon and Schiestl, 2021). Additionally, linkage disequilibrium (LD) has been observed to decay more rapidly with higher rates of outcrossing due to increased effective recombination (Morrell et al., 2005; Zhu et al., 2016), with self-incompatibility (i.e., forced outcrossing) leading to even more rapid decay as observed in the tea plant (*Camellia sinensis* [L.] Kuntze; Theaceae; Niu et al., 2019). Bird pollination may increase reproductive success as compared to bee pollination (Abrahamczyk et al., 2022), with bird pollination possibly leading to an increase in the rate of outcrossing (Whelan et al., 2009) and thus increased LD decay. Given that positive selection on certain traits can also result in elevated LD, as observed in a floral homeotic mutant of *Aquilegia* L. (Ranunculaceae; Cabin et al., 2022), measures of LD across closely related species can indicate genomic-scale selection in action.

In this study we provide a genome-wide investigation into the genetic underpinnings of morphological traits associated with shifts in pollinators, as well as conducting a deeper investigation into many candidate genes associated both with specific floral traits and with correlated trait evolution resulting in floral syndrome phenotypes. Specifically, we set out to: (1) provide an updated phylogenetic hypothesis from a comprehensive sampling of the diversity of Neotropical *Costus*, using a target enrichment approach with custom designed genomic baits (Valderrama et al., 2020). We increased the sampling of *Costus* diversity over previous studies by 84 accessions to further test species delimitations, associate biogeographic patterns with morphologic diversity including polymorphism within species, and estimate relationships for newly described taxa. We inferred a robust phylogeny for 66 species (represented by 70 OTUs) using concatenation and coalescent-based species tree methods. (2) Investigate morphological changes associated with pollination syndromes across the genus, to test if inflorescence traits associated with pollination and/or defense against herbivory are constrained by the phylogenetic relationships of Neotropical *Costus* species, and if those traits evolved in a correlated manner. Furthermore, we test if different diversification rates are attributable to traits related to pollination syndromes. (3) Identify signatures of selection across putative neutral loci and candidate genes underlying morphological changes. Genes of interest were identified in the literature as influential for floral development and associated with traits that together generate classic pollination syndromes to test for structural variation associated with “mellitophilous” or “ornithophilous” flowers across the diversity of Neotropical *Costus*. (4) Compare genome-wide signatures across different pollinator types. We obtained Illumina short reads from whole genome sequencing for a subset of 20 closely related species with contrasting (bee vs. hummingbird) pollination syndromes. These data were used to identify gene regions that could be associated with corresponding shifts of floral morphology that were not included in the target bait set; the genome-wide data were also used to investigate relationships among targeted taxa outside of a purely bifurcating framework using a phylogenetic network given that some were of known hybrid origin.

## Materials and methods

The phylogenomic approach presented here is based on previous work using a targeted enrichment strategy designed to capture nuclear loci specifically for *Costus*, resulting in a robust topology of the Neotropical *Costus* radiation (Valderrama et al., 2020). Targeted regions were selected using genome assemblies for *Costus spicatus* (Jacq.) Sw. and *Costus longibracteolatus* Maas submitted to the Phyluce pipeline (Faircloth et al., 2012; Faircloth, 2016) with customizations to avoid repetitive and potentially non-homologous regions, removal of plastid genomes and maintaining GC content of the regions between 37 and 55% (details in Valderrama et al., 2020). The baits included target loci identified in previous studies as being single-copy and useful for phylogenomic inferences within the order Zingiberales (240 regions from Sass et al., 2016; 47 regions from Carlsen et al., 2018) as well as 235 loci for genes known to be involved in the evolution of floral development, yielding a total bait set of 1,521 loci and over 1 million base pairs. Custom probes were synthesized in 100 mers with a 20 K design by myBaits (Arbor Biosciences, Ann Arbor, MI, United States) with 3× tiling. Sampling was expanded from Valderrama et al. (2020) to include an additional 31 species (distributed in 76 accessions) of the Neotropical radiation of *Costus* for a total of 61 species (171 accessions) of the c.77 described species (Supplementary Table 1). These accessions were selected to represent morphologic and biogeographic variation within described species. Additionally, 48 accessions representing the major African *Costus* lineages and Neotropical Costaceae were included as outgroups.

### Extraction and library prep

DNA was extracted from either silica-dried leaf material collected in the field or from herbarium specimens with protocols that use SDS detergent (Edwards et al., 1991; Konieczny and Ausubel, 1993). Size distributions of the extracted fragments were visualized on a 1% agarose gel and concentrations were quantified with a Qubit 3.0 Fluorometer (Life Technologies, Grand Island, NY, United States). When the majority of fragments for any given sample were longer than 350 bp, a Covaris E220 evolution Focused-ultrasonicator (Covaris, Woburn, MA, United States) was used following manufacturer’s protocols to obtain an average fragment size of 350 bp. Double-sided size selection was performed with size selection beads using a homemade solution of Carboxyl-modified Sera-Mag Magnetic Speed-beads (Thermo Fisher Scientific, Freemont, CA, United States) in a PEG/NaCl buffer (Rowan et al., 2017). The size selection protocol was simplified to only discard smaller fragments for samples from herbarium specimens that yielded low DNA concentrations largely comprising short fragments.

Dual-indexed Libraries were prepared with the KAPA Hyper Prep kit following the manufacturer’s recommendations with 500 ng of DNA fragments around 350 bp in length. Modifications were made to reduce the volume of the reactions to 1/5th of the manufacturer’s protocol shared by Lydia Smith at the Evolutionary Genetics Laboratory at UC Berkeley (protocol^1^). Indexed libraries were pooled (6–10 libraries per reaction) and enriched following the manufacturer’s instructions (myBaits Manual v4.01, Arbor Biosciences, Ann Arbor, MI, United States) with a hybridization temperature of 65°C for 24 h. To improve the performance of the blocking oligos, the mix supplied with the baits (i.e., Roche Universal Blocking Oligo Kit and SeqCap EZ Developer Reagent) was substituted with plant C0t-1 DNA (Portik et al., 2016). Pooled libraries were enriched, amplified and sequenced on a lane of NovaSeq SP 150 PE in the Vincent J. Coates Genomics Sequencing Laboratory at UC Berkeley after final quantification and pooling.

### Assembly and alignment

Low quality bases as well as adapters were removed from the reads using TrimGalore^2^ (Martin, 2011) and normalized to 100× coverage using bbnorm within the BBtools package.^3^ Reads were mapped and assembled to the 1,521 target loci using HybPiper v1.3.1 (Johnson et al., 2016) with default settings. Paralog sequences for the assembled loci were retrieved with HybPiper. The resulting contigs were combined with data from Valderrama et al. (2020) and loci with paralog warnings obtained for more than 5% of the accessions in recovered loci were excluded from downstream analyses. Alignments were estimated with MAFFT v7.271 (Katoh and Toh, 2010) with the iterative refinement method incorporating local pairwise alignment information and with a gap opening penalty of 10 (maximum iterations set to 10,000) and poorly aligned bases and spurious sequences removed with Trimal v1.4.1 (Capella-Gutiérrez et al., 2009) with the automated1 option. Only exonic sequences were used because including introns resulted in poor alignments.

### Phylogenomic inference

Gene trees were estimated using IQTree v2.1.2 (Minh et al., 2020), using the ModelFinder (Kalyaanamoorthy et al., 2017) option (-m MFP) to find the best DNA substitution model followed by maximum likelihood inference to generate a tree for each partition. TreeShrink v1.3.7 (Mai and Mirarab, 2018) with default values for the species mode (*a* = 0.05, *b* = 5%) was used to detect irregularly long branches in the gene trees. The TreeShrink algorithm identifies significantly long branches and removes them in the respective trees and alignments using the distribution of branch lengths for each individual within the gene trees. All loci purposed for phylogenetic inference (excluding the floral development genes) were concatenated and a GTR + G substitution model was fit to each gene. Before finding the best tree, partitions corresponding to each gene were merged using the greedy heuristic algorithm (Lanfear et al., 2014). To prevent the exploration of partition merging schemes from becoming computationally intractable, the relaxed cluster algorithm (rcluster option; Lanfear et al., 2014) was set to examine only the top 10% of the schemes. The ultrafast bootstrap approximation (Hoang et al., 2018) combined with the single branch SH-like approximate likelihood ratio test (Guindon et al., 2010), each with 10,000 replicates, was used to assess support for the resulting branches.

To account for discordance among gene trees, the species tree was estimated with ASTRAL v5.6.3 (Zhang et al., 2018b) using gene trees obtained from IQtree for each locus. The R packages treeio v1.10.0 (Wang et al., 2020) and ggtree v2.0.4 (Yu et al., 2017) were used to plot the quartet support values estimated with ASTRAL on the resulting topology using the −t2 output option. Phytools v0.7-90 (Revell, 2012) function cophylo was used to compare the concatenation and coalescent-based species trees.

### Trait scoring and frequency

Features of the inflorescences identified to be related to pollination syndromes were scored as binary traits for all included taxa, using published literature for recognized species and including data from proposed species to be included in an updated monograph of the new world Costaceae (Maas, 1972, 1977; Maas et al., submitted^4^; Supplementary Table 2). The traits corresponding to pollination syndrome are: [1] **labellum shape** (tubular/spreading), which directly corresponds to observations of flower visitors, with hummingbirds visiting flowers with a tubular labellum and bees visiting flowers with an horizontally spreading labellum (Kay et al., 2005); [2] **bract color** (red/green), including those with bright orange or yellow bracts in the red category, e.g., *Costus lasius* Loes. and *Costus wilsonii* Maas; [3] **bract appendages** (present/absent), with four species (*Costus barbatus* Suess., *Costus callosus* Maas & H.Maas, *Costus curvibracteatus* Maas, and *Costus juruanus* K.Schum) recognized as having extensions of the bract apex that resemble appendages but are not exactly the same anatomical structure described by Maas (1972). Because extensions can play a similar role in the appearance of the inflorescence, species with extensions were coded as present for appendages; [4] **“honey mark”** or nectar guide (present/absent), as a colored mark located in the center of the labellum; and [5] **labellum stripes** (present/absent), with those coded as present having lateral markings across the labellum that contrast with the ground color. In addition, we also coded a trait that indicated the overall appearance of the inflorescence, [6] **functional color** (red/non-red). In many cases, the color of the bracts is completely hidden by the presence of appendages (e.g., *Costus leucanthus* Maas or *Costus sinningiiflorus* Rusby) such that the inflorescence presents as the color of the appendages. This is likely to influence interactions with pollinators, for example creating a “functionally green” inflorescence even though bracts are scored as red. In this case, the functional color is coded as non-red to reflect the color of the appendages.

Additional traits potentially associated with defense against herbivory, but also relevant for pollination, are: [7] **callus** (present/absent), referring to the extrafloral nectar producing gland located on the abaxial side of inflorescence bracts; and [8] **inflorescence indument** (present/absent), referring to the presence or absence of indument on bracts and/or bract appendages. All polymorphic characters were coded as ambiguous (both states) and our scoring represents the known variation within each species.

The UpsetR v1.4.0 package (Lex et al., 2014) was used to visualize the subset of observed combinations of characters found in our sampling and the frequency of those combinations. Traits were variable for some taxa and the weight was divided into the possible combinations within each lineage (maximum of four combinations due to two variable traits). To prevent including biologically unrealistic combinations, all combinations of characters that were only observed because two traits were polymorphic for the same lineage were removed. Also, four lineages with three ambiguous/polymorphic characters each (*C. arabicus* L., *Costus guanaiensis* Rusby, *Costus lima* K.Schum., and *C. longibracteolatus*) were removed from the analysis.

### Comparative methods

**FIGURE 1:**
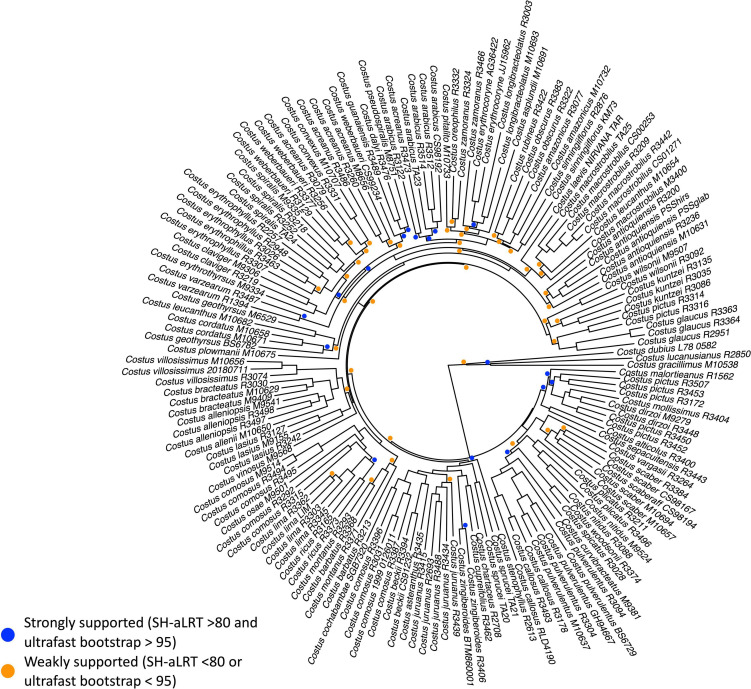
Pruned maximum likelihood (IQtree) concatenation phylogeny of 945 loci. The values above the branches are the result of the SH-aLRT (above 80 are considered strongly supported) and ultrafast bootstrap support (above 95 are considered strongly supported) showing high support values in most of the branches. Support values are 100/100 unless otherwise specified. Well-supported SH-aLRT (>80) and ultrafast bootstrap (>95) are denoted with a blue circle, while support values of either SH-aLRT or ultrafast bootstraps lower than the strongly supported cutoff are denoted with an orange circle. African *Costus* and Neotropical Costaceae accessions used as outgroups are not shown.

Individuals of the same species that formed monophyletic groups were pruned to leave a single accession per species. *Costus acreanus* (Loes.) Maas, *C. comosus* (Jacq.) Roscoe, and *C. leucanthus* accessions were non-monophyletic (see Figure 1 and section “Results”), therefore a single accession was kept per clade (or lineage) to encompass the evolutionary history within each named taxon. Before applying comparative methods, the phylogenetic signal of each analyzed trait was estimated with the D statistic of phylogenetic dispersion (Fritz and Purvis, 2010). Both null hypotheses of the characters evolving following a random (no phylogenetic signal) or a Brownian model (strong phylogenetic signal) were assessed for each binary character (100,000 permutations) using the phylo.d function of the R package caper v1.0.1 (Orme et al., 2013). To reconstruct ancestral character states, stochastic character mapping (Huelsenbeck et al., 2003) was used with models including equal and different transition rates for each binary trait using the make.simmap function of phytools v0.7-90 (Revell, 2012). One thousand stochastic character maps were summarized to estimate posterior probabilities of the ancestral states of each character. The equal and different rate models were compared with a likelihood-ratio test. Because most of the comparative methods require ultrametric trees, the phylogenetic tree from the concatenated analysis was transformed using penalized likelihood with correlated rate variation among branches (Kim and Sanderson, 2008) using the chronos function in the R package ape v5.5 (Paradis and Schliep, 2019).

The Pagel (1994) analysis of correlated evolution was used to test if models of independent evolution vs. dependent evolution were more appropriate for each possible pair of traits given our data; analyses were run with the fitPagel function (fitMk method) available in the R package phytools. Models with equal and different transition rates were tested for each comparison. A Bonferroni (1935) correction was applied to the *p*-value corresponding to the Chi-squared test of the likelihood ratio test to account for the inclusion of each trait in seven comparisons.

To explore if diversification rates are different between lineages with different pollination syndromes, models with constant diversification rates vs. Binary State Speciation and Extinction (BiSSE; Maddison et al., 2007) models were compared with different diversification parameters set for lineages with tubular vs. spreading labellum (a proxy for ornithophilous and melittophilous pollination syndromes). These results were compared to Hidden State Speciation and Extinction models (HiSSE; Beaulieu and O’Meara, 2016) that account for potential distinct diversification regimes unrelated to pollination syndromes by fitting three distinct models, one with a hidden trait accompanying pollination syndromes and two pollination-independent models. For the two trait-independent models, the first included two hidden states (CID-2 HiSSE model) and the second included four hidden states (CID-4 HiSSE model). All models were fitted with the hisse v2.1.6 R package (Beaulieu and O’Meara, 2016) keeping extinction parameters linked.

### Transcriptome analyses

Fresh material (whole flowers, inflorescences, and leaves for *C. spicatus*) was flash frozen using liquid nitrogen followed by storage at −80°C. Total RNA was extracted following the protocol of Yockteng et al. (2013), cleaned with homemade ampure beads (Rowan et al., 2017), and quantified with a Nanodrop and Qubit 3.0 Fluorometer (Life Technologies, Grand Island, NY, United States). For short-read Illumina sequencing, RNA was sent to the Vincent J. Coates Genomics Sequencing Laboratory at UC Berkeley (Berkeley, CA, United States) for their full service library preparation method using ribodepletion and sequencing on an Illumina NovaSeq 2 × 150. Raw Illumina reads were adaptor-trimmed and filtered for low quality bases using fastp v0.20.1 (Chen et al., 2018). Long reads for leaf and flower RNA were generated for cDNA libraries constructed using the cDNA-PCR Barcoding library kit (SQK-PCS109 and SQK-PBK004) with 100 ng of total RNA for each sample and sequenced on a single Oxford Nanopore minION flow cell. The resulting fast5 files were used to call fastq files using guppy basecaller v3.4.1 (Oxford Nanopore) with a minimum quality score of seven. Fastq files were then demultiplexed and adaptor-trimmed using porechop v0.2.4.^5^ Nanopore reads were error corrected prior to transcriptome assembly using FMLRC v1.0.0 (Wang et al., 2018) after first building a FM index using the cffq function in MSBWT v0.3.0 (Holt and McMillan, 2014). The error-corrected reads were then filtered using seqkit v0.12.0 (Shen et al., 2016) to discard reads shorter than 800 bp.

A *de novo* transcriptome assembly was generated to serve as a reference to identify candidate genes of interest for downstream analyses using Trinity v2.11.0 (Grabherr et al., 2011; Haas et al., 2013) using both the cleaned Illumina short reads and Nanopore long reads by implementing the long-reads flag. This approach has been used previously in several studies to generate full length or nearly full length gene contigs in the reference transcriptome assembly to map Illumina short reads to for differential gene expression analysis (Landis et al., 2020; Zhang et al., 2022). Contigs smaller than 250 bp in length were removed with seqkit v0.12.0 (Shen et al., 2016). The assembled transcriptome was annotated using Trinotate v3.2.2 (Bryant et al., 2017) and Transdecoder v5.5.0 (Haas et al., 2013). Contigs were compared to the SwissProt database with ncbi-blast v2.5.0 (Camacho et al., 2009) using BLASTX for nucleotide sequences of contigs and BLASTP for translated amino acids of each contig. Completeness of the assembled transcriptome was determined by using the embryophyta Benchmarking Universal Single-Copy Orthologs (BUSCO) v3.0.2 (Simão et al., 2015) and summarized in terms of number and size of contigs with NanoPlot (De Coster et al., 2018).

### Genome sequencing and analysis

A reference draft genome of *Costus bracteatus* Rowlee was assembled using both Illumina short reads and Nanopore long reads to be used as reference for SNP calling for downstream analyses. Fresh leaf material of *C. bracteatus* R3030 was collected and immediately shipped on ice overnight to California State University East Bay. Leaves were stored at −80° upon arrival until further use. DNA for Illumina sequencing was extracted using the GeneJET Plant Genomic DNA Purification Mini Kit (Thermo Fisher Scientific) following the manufacturer’s instructions as described in Grunder (2021) and quantified using a NanoDrop (Thermo Fisher Scientific). DNA was sent to the Vincent J. Coates Genomics Sequencing Laboratory at UC Berkeley (Berkeley, CA, United States) for library preparation following in-house DNASeq protocol for the Illumina NovaSeq 2 × 150 to generate approximately 80 GB of sequencing data. Raw Illumina sequencing reads were adaptor-trimmed and filtered for low quality bases using fastp v0.20.1 (Chen et al., 2018). Due to the read depth generated and the unevenness of sequencing across the genome, the cleaned reads were normalized to 100× coverage using the bbnorm function in BBtools (see text footnote 3). Illumina genome sequencing was generated for an additional 19 accessions of *Costus* (Supplementary Table 3) following the same extraction and sequencing protocols. A total of 80 GB of data was targeted for each accession and processed using the same methods described above. The 20 species used for Illumina sequencing were initially identified as sister species with different pollination syndromes. Unfortunately, the most recent phylogeny of Valderrama et al. (2020) showed that many of these species were no longer sister species. However, the genomic data generated still allows insights into genetic changes associated with different pollinator syndromes.

DNA for Nanopore sequencing was extracted using a modified SDS protocol with ampure beads (Schalamun et al., 2019). After extraction, the DNA was further cleaned with a 24:1 chloroform:isoamyl alcohol step, followed by ethanol precipitation with ammonium acetate. Cleaned DNA was then passed through the Qiagen DNAEasy PowerClean Pro Cleanup Kit (Qiagen) and resuspended in 50 μL of sterile water. Concentration and quality of the final DNA was performed on a Nanodrop. Genomic Nanopore libraries were constructed using the Genomic DNA by Ligation kit (SQK-LSK109) using 1–1.5 μg of clean DNA as template for library preparation. Each completed library was sequenced on a minION flow cell for 72 h. A total of three flowcells were sequenced, generating 39.34 GB of raw sequencing data. Fast5 files were basecalled with a GPU instance of guppy basecaller v6.0.0 + ab7925058 (Oxford Nanopore) with a minimum quality score of five. Fastq files were then adaptor trimmed using porechop v0.2.4 (see text footnote 4). Nanopore reads were error corrected prior to genome assembly using FMLRC v1.0.0 (Wang et al., 2018) after first building a FM index using the cffq function in MSBWT v0.3.0 (Holt and McMillan, 2014). A hybrid genome assembly approach was undertaken with MaS uRCA v3.4.2 (Zimin et al., 2013) to utilize both the Illumina short reads and the error-corrected Nanopore long reads. Default parameters were used in the assembly except the inclusion of 35× coverage of the long reads. The final assembly was polished using POLCA (Zimin and Salzberg, 2020) as implemented in MaSuRCA v3.4.2. Without flow cytometry analyses, the genome size of *C. bracteatus* was estimated using GenomeScope (Vurture et al., 2017) with the Illumina reads as input.

As a way to assess the quality and completeness of the MaSuRCA hybrid genome assembly approach, the adaptor-trimmed Nanopore long reads were used to assemble the genome of *C. bracteatus* with Flye v2.8.3-b1705 (Kolmogorov et al., 2019). The subsequent assembly was polished with the Illumina reads using POLCA as described for the MaSuRCA assembly. The completeness of both genome assemblies was determined by using the embryophyta BUSCO v3.0.2 (Simão et al., 2015) and summarized in terms of number and size of contigs with NanoPlot (De Coster et al., 2018).

### Selection analyses

To assess the evolutionary implications of candidate genes associated with phenotypic traits, especially given the lack of quantitative values of gene expression, signatures of selection were analyzed using codeml (Jeffares et al., 2015) in PAML v4.9 (Yang, 2007). Two different data sets were used: a subset of nearly 300 loci from the full capture set and a smaller data set comprising candidate loci generated from the 20 accessions of Illumina genome sequencing. For the larger data set, the longest open reading frame was first extracted for all 1,472 loci that were retrieved from HybPiper and pruned as described above using TransDecoder v5.5.0. The resulting fasta file was then aligned using MAFFT v7.271 (Katoh and Standley, 2013) using the auto and adjustdirectionaccurately flags, followed by trimming with TrimAL v1.4.1 (Capella-Gutiérrez et al., 2009) with the automated1 option. This filtering left nearly 300 loci, which were checked for the appropriate reading frame using AliView v1.28 (Larsson, 2014). Premature stop codons were replaced with NNN using the exportAlignment function in MACSE v1.2 (Ranwez et al., 2011). The final fasta file for each locus was used to generate a gene tree with RAxML v8.2.12 (Stamatakis, 2014) with 100 rapid bootstrap replicates under a GTR + G model of molecular evolution. For each locus with a cleaned alignment and gene tree, a summary omega value (dN/dS) was calculated with codeml using model = 0.

For the smaller data set, candidate genes associated with flower color, size, symmetry, and fusion were identified from the annotated transcriptome that was assembled for *C. spicatus* (see above). Contigs for known candidate genes associated with flower size included *BIG BROTHER* (Disch et al., 2006), *GASA/GAST1/GEG* (Shi et al., 1992; Herzog et al., 1995; Kotilainen et al., 1999), *LONGIFIOLIA* (*LNG*; Lee et al., 2006), and *AUXIN RESPONSE FACTOR 2* (*ARF2*; Ellis et al., 2005). Genes associated with symmetry include *RADIALIS* (Coen et al., 1995; Boyden et al., 2012) and *DIVARICATA* (Almeida et al., 1997). Genes associated with fusion include *AGL6* (Koo et al., 2010), *CUP SHAPED COTYLEDON* (Aida et al., 1997), *PETAL LOSS* (*PTL*; Lampugnani et al., 2012), *RABBIT EARS* (Krizek et al., 2006), *SUPERMAN* (Sakai et al., 1995), and *YABBY* (Ha et al., 2010). Genes associated with flower color include chalcone synthase (*CHS*), dihydroflavonol 4-reductase (*DFR*), flavonol synthase (*FLS*), and flavonoid 3′-hydroxylase (*F3′H*) (Holton and Cornish, 1995). A fasta file of contigs for each of the above genes was used as a reference file during mapping of the genomic Illumina data for 20 species using bwa-mem2 v2.2.1 (Vasimuddin et al., 2019). The resulting SAM files were converted to BAM and then sorted using Samtools v1.12 (Li et al., 2009). The sorted BAM files were then split using BamTools v2.5.1 (Barnett et al., 2011) so that each candidate gene was in a separate file. Consensus sequences for the coding region of each candidate gene was then generated using the dark-matter^6^ python script make-consensus.py which utilized bcftools v1.9 (Li, 2011) and iVar v1.3.1 (Grubaugh et al., 2019). The resulting fasta files for each locus were combined into a single fasta file, followed by alignment with MAFFT, cleaning with TrimAL, removal of premature stop codons, and gene tree inference as described for the large data set. Due to most gene sequences possessing polymorphic bases represented with ambiguity codes, the PAML settings were changed to remove ambiguous sites. For each locus, a summary omega value (dN/dS) was generated with codeml using model = 0 and an omega value for every branch was generated with model = 1. Omega values less than one indicate purifying selection, omega values equal to one indicate neutral evolution, and omega values above one indicate positive selection.

### Single nucleotide polymorphism calling analyses

To investigate genomic changes associated with different pollinators, and to more fully explore the evolutionary history of a subset of taxa, SNPs were called for the 20 species with Illumina genome sequencing data. Cleaned reads were mapped to the *C. bracteatus* polished MaSuRCA assembly using bwa-mem2 v2.2.1 (Vasimuddin et al., 2019). Resulting SAM files were converted to BAM and then sorted using Samtools v1.12 (Li et al., 2009). Duplicates were marked using piccard v2.21.2 (Broad Institute, 2019). For each species, SNPs were first called using HaplotypeCaller in GATK v4.1.4 (McKenna et al., 2010) using the ERC flag to create a GVCF (Genomic Variant Call Format) for each species. All GVCFs were then combined using the GenomicsDBImport followed by joint genotyping using GenotypeGVCFs. Resulting VCF (Variant Call Format) files were then combined into a single VCF using the GatherVcfs function.

The VCF file was initially filtered in VCFtools v0.1.16 (Danecek et al., 2011) using the following parameters: biallelic sites with a maximum missing 0.9, minimum mean depth of 15, maximum mean depth of 200, minimum quality score of 30, minor allele frequency of 0.05, and minor allele count of 3. LD was calculated using PopLDdecay (Zhang et al., 2018a) on the initial filtered VCF for all samples, ornithophilous species only, and mellitophilous species only. The VCF file was further filtered by LD pruning using plink v1.90b6.21 (Purcell et al., 2007) using a *r*^2^ of 0.2 using a 20 kb window with a 10 kb step size. A phylogenetic network was generated in SplitsTree v4 (Huson and Bryant, 2006) using the final filtered VCF file containing 3,432,721 SNPs after converting to a nexus format with vcf2phyllip (Ortiz, 2019). A phylogenetic network allows for conflicting splits due to gene flow, which we know exists due to our sampling of *Costus vinosus* Maas × *allenii* Maas R3353 and *C. wilsonii* × *villosissimus Jacq.* “mellow yellow” R3091, both being suspected hybrids sampled from plants in cultivation (D. Skinner, personal observation). In addition to the phylogenetic network, a principal components analysis (PCA) was conducted using the R package SNPrelate v3.14 (Zheng et al., 2012) coloring species as bird pollinated, bee pollinated, or known hybrids. The first four principal components (PC) were plotted. Lastly, the level of heterozygosity from the final pruned SNP data set was calculated for the three groups: bee pollinated, bird pollinated, and known hybrids using VCFtools v0.1.16 (Danecek et al., 2011).

## Results

### Phylogenetic relationships

The capture approach generated 945 loci for a total alignment of 751,609 bp with 355,314 distinct patterns, 125,357 singleton sites and 425,080 constant sites, for 171 accessions representing 61 of a total of ca. 77 species of the Neotropical radiation of *Costus* (see Supplementary Table 1). The topology obtained with the concatenation approach is well supported with most branches showing high support values with the SH-aLRT (>80) and ultrafast bootstrap (>95) (Figure 1 and see Supplementary Figure 1 for the full phylogeny including outgroups). These values are comparatively lower for the lineages comprising the earliest branches of the Neotropical *Costus* radiation. The species trees approach implemented in ASTRAL produced a similar topology but with some differences in the earliest branches (Supplementary Figure 2). The normalized quartet score of the topology obtained with ASTRAL is 46.82%, suggesting high levels of discordance among gene trees. The quartet scores indicate high levels of gene tree conflict in less supported branches and some of the branches with high local posterior probabilities show several gene trees supporting the alternative topologies of each quartet (Supplementary Figure 3). The same well-supported lineages were recovered with both methods and we used the topology inferred from the concatenation approach in subsequent analyses. Most species that were sampled for more than one individual are recovered as monophyletic in the resulting phylogeny, even when considering broad geographic variation or morphological variation. However, some accessions determined as conspecifics comprise independent lineages, such as individuals from western Mesoamerica identified as *Costus pictus* D.Don, that are not related to the accessions of individuals identified as belonging to the same species from eastern Mesoamerica. Our sampling of *C. comosus* and *C. acreanus* reveals at least three independent lineages included in each species, suggesting the presence of species complexes. Similarly, *C. leucanthus* is represented by two lineages highlighting additional hidden diversity within the genus.

### Traits combinations and comparative methods

**FIGURE 2:**
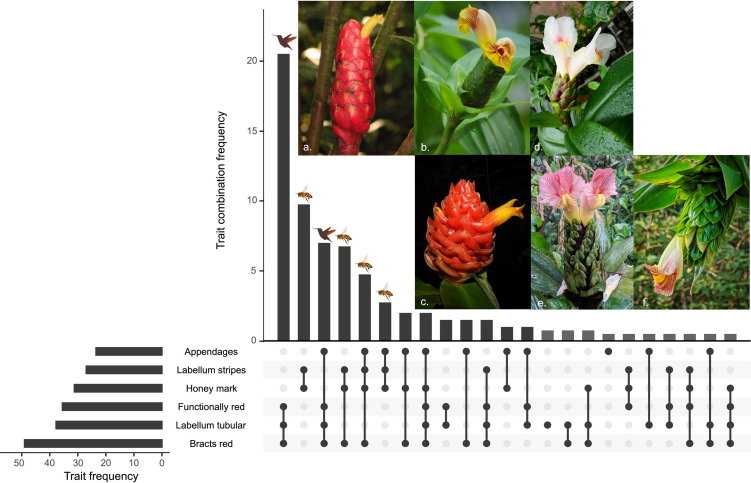
Frequency of the analyzed traits and their combinations in our sampling. Traits were variable for some taxa and the weight was divided into the possible combinations within each lineage (maximum four combinations due to two variable traits). Gray bars represent combinations only observed in lineages with polymorphic traits (combinations only observed because of the permutation of two polymorphic traits were excluded). Ornithophilous and mellitophilous syndromes are indicated with the bee or hummingbird icons when trait combinations match all the expectations of the pollination syndromes. Examples of species showing the first six trait combinations are shown in the photographs corresponding to the following species: **(a)** *Costus plowmanii* Maas, **(b)** *Costus dirzoi* García-Mend. & G.Ibarra, **(c)** *Costus ricus* Maas & H.Maas, **(d)** *Costus acreanus* (Loes.) Maas, **(e)** *C. sinningiflorus* Rusby, **(f)** *C. bracteatus* Rowlee. All photos by D. Skinner except *C. plowmanii* by P.J.M. Maas and *C. ricus* by Reinaldo Aguilar.

The most prevalent combinations of the analyzed traits match expectations of pollination syndromes (Figure 2). The most frequent combination of traits corresponds to a morphology associated with hummingbird pollination with a tubular labellum (without stripes or honey mark), overall red inflorescences, and no appendages on the bracts (Figure 2a). The second most observed combination agrees with bee-pollinated inflorescences, with a spreading labellum (with stripes and honey mark) and green bracts, and no appendages on the bracts (Figure 2b). The third and sixth most common combinations correspond to hummingbird and bee-pollinated morphologies, respectively, but with appendaged bracts (Figures 2c,f). The fourth most frequent combination of traits matches bee pollination with spreading labellum (with stripes and honey mark) and red bracts, but with functionally non-red inflorescences, the tips of the bracts are green in color (Figure 2d). Red bracts can also be concealed by the green and leafy appendages of the bracts as in the fifth most prevalent combination (Figure 2e), seen in *C. bracteatus* and *C. sinningiiflorus*, making this morphology functionally identical to that of the sixth most-common combination (compare Figures 2e,f). The other observed combinations do not match all the expectations of pollination syndromes but are less frequent and constitute the tail of a positively skewed distribution.

Comparing the empirical D statistic for each trait with permutations cannot reject the hypothesis of the traits evolving under a Brownian motion model (or with a strong phylogenetic signal) (Supplementary Table 4). The hypothesis that the analyzed traits evolved randomly or with no phylogenetic signal was rejected for all traits (*p* < 0.05) with exceptions for bract color, presence/absence of appendages in the bracts and presence/absence of the callus. The presence or absence of the callus in the bracts is the only trait where the D statistic value is closer to the ones obtained under a model of random evolution (with no phylogenetic structure). The all-rates-different model significantly improved the likelihood scores for how the models fit the transitions of the labellum shape, labellum stripes and appendages in the bracts. For functional color, bract color, presence of the honey mark, callus and inflorescence indument, an equal rates model was favored (Supplementary Table 4).

**FIGURE 3:**
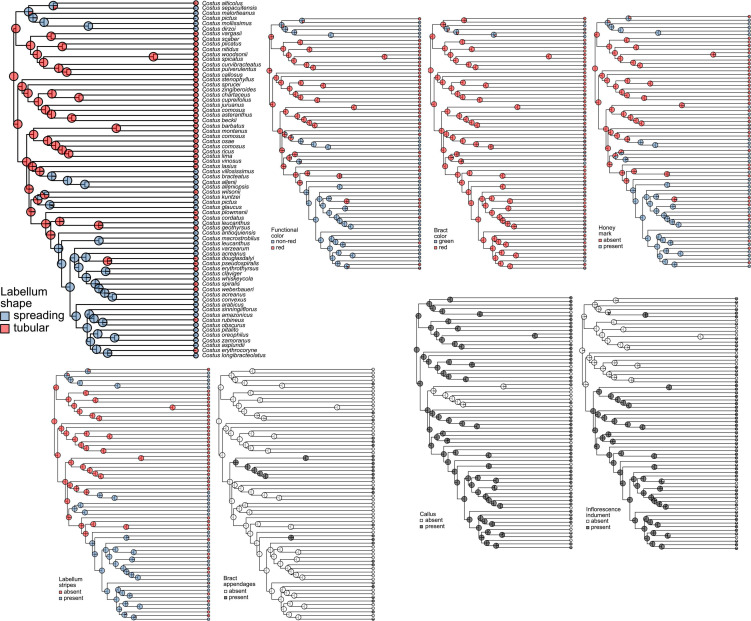
Summary of the stochastic character mapping of the evolution of pollination and defense against herbivory traits in Neotropical *Costus*. Pie charts indicate the posterior probabilities obtained from the 1,000 stochastic mappings. Melittophily associated trait states are shown in blue and ornithophily associated trait states in red. Presence of traits associated with defense against herbivory are shown in gray and absence in white.

The stochastic character mapping of the labellum shape indicates that the tubular shape is the most likely ancestral state of Neotropical *Costus* and that the spreading labellum evolved twice and subsequently reverted to tubular shape in several instances (Figure 3; Labellum shape). The other characters related to pollination appear to shift after changes in labellum shape, following the expectations of trait combinations that constitute pollination syndromes. Lineages gaining a spreading labellum subsequently become green in their inflorescence color, acquire labellum stripes, and acquire a honey mark (see Figure 3). The stochastic mapping of the bract appendages does not show the same pattern and suggests that these structures have evolved multiple times in several lineages from ancestors without appendages, independently of other traits and of pollination syndrome. The callus in the bracts has been lost in several lineages independently, while the inflorescence indument has been gained independently in several instances (Figure 3).

**FIGURE 4:**
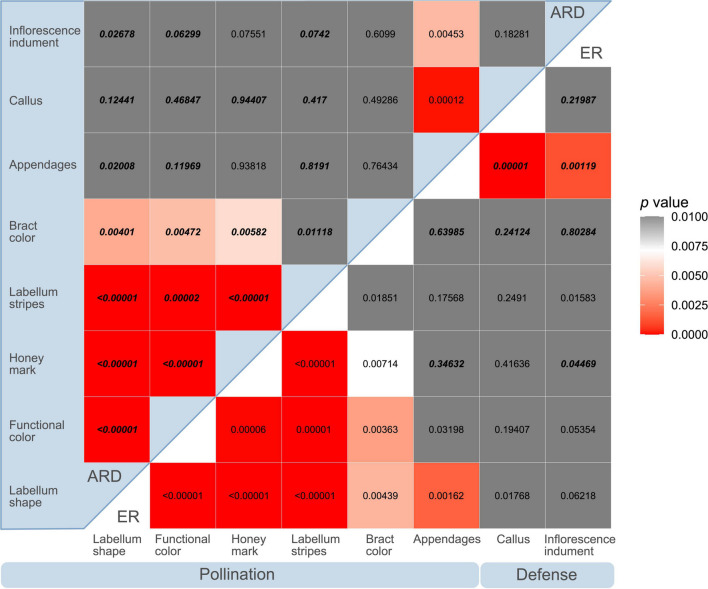
Heatmap showing the *p*-values for the independent evolution model based on a Chi-squared test to compare with Pagel’s model of correlated evolution of traits associated with pollination or with defenses against herbivory. Results are shown for equal transition rates (ER) and all rates different (ARD) models for each possible pair of traits. The *p*-value of the model with lower AIC value for each pair is shown in bold, bigger and italicized font. The color scale was adjusted to reflect the correction for multiple comparisons with white cells for the values close to the significance threshold (0.007143); red cells indicate significant *p*-values and correlated evolution and gray cells non-significant values and independent evolution of the pair of traits.

**FIGURE 5:**
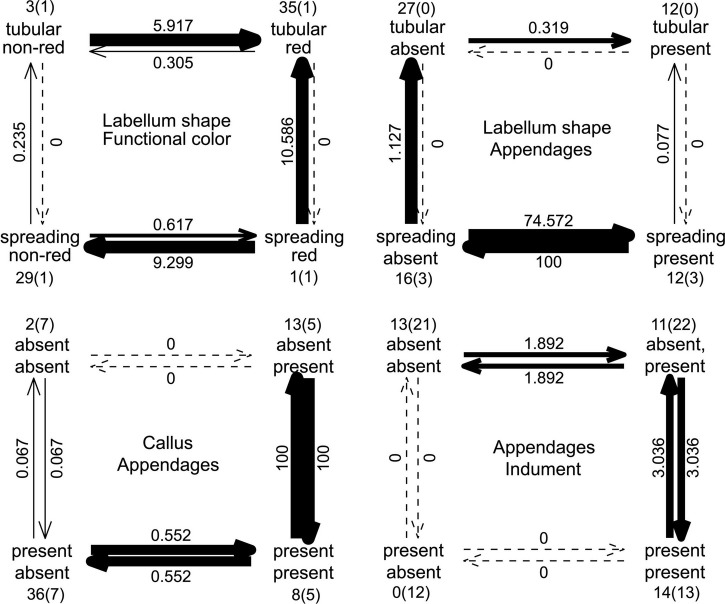
Plot showing the transitions between the possible combinations of the character states in the models of correlated evolution for different pairs of traits. Thickness of the arrows is proportional to the values of the rates. Number of observations for each combination of characters are indicated. The number of additional observations involving a species presenting both states of one of the characters are shown in parentheses.

The Pagel analyses of correlated evolution show that pollination related traits (labellum shape, functional inflorescence color, honey mark, and labellum stripes) are strongly correlated (*p* < 0.00001) with bract color also correlated (*p* < 0.004) (Figure 4). The labellum shape, thought to be the most important character for pollination syndrome, is only marginally correlated with the evolution of appendages in the bracts. The evolution of the callus and the inflorescence indument, which potentially play a role in defense against herbivory, are not correlated with each other or with the pollination traits, but are both strongly correlated with the presence of appendages on the bracts (Figure 4). The transition rates for the model of correlated evolution of the labellum shape and the functional color of the inflorescence indicate that the most stable combinations of characters match the pollination syndromes, with the alternative combinations transitioning promptly to a tubular labellum shape and red inflorescence or to a spreading labellum and non-red inflorescence (Figure 5). Note that a non-red functional color can be obtained either by changing to a green bract color or by gaining leafy appendages. In the model of correlated evolution of the labellum shape and the bract appendages, the transition rates of the appendages are higher in the lineages with a spreading labellum, suggesting that this trait is more labile in the lineages with this labellum shape (which is also associated with bee pollination). Similarly, when appendages are present, the transition rates for gaining or losing the callus in the bracts are higher. The lineages with the indument on the inflorescence also show higher transition rates for the presence or absence of the bract appendages (Figure 5).

The model with constant diversification rates has the lowest fit when comparing AICc values. Models with hidden states that are independent of the labellum shape have the best fit, therefore we found no evidence of differential diversification rates related to the labellum shape and consequently to the pollination syndromes (Supplementary Table 5). The CID4 model had a deltaAIC of 18.21 to the second closest model of the full HiSSE model.

### Transcriptome and genome assemblies

After quality filtering and adaptor trimming of the RNA-Seq raw Nanopore reads, a total of 2,291,573 reads with a N50 of 818 bp resulted for the *C. spicatus* whole flower and 2,605,893 reads with a N50 of 822 bp for the leaf material. Illumina sequencing generated 104 million reads for the flower material and 80 million reads for the leaf material (Supplementary Figure 4a). The Trinity transcriptome assembly generated 227,713 contigs with an N50 of 1,795 bp. According to the embryophyta BUSCO library, the *C. spicatus* transcriptome contained 1,335 out of 1,375 complete BUSCOs, with 560 represented as single-copy and 775 represented as duplicated. The transcriptome assembly had 18 fragmented BUSCOs and 22 missing BUSCOs.

After filtering, adaptor trimming, and normalization of the genome Illumina data for *C. bracteatus* a total of 133,777,645 reads representing approximately 40 GB of data remained. After filtering the Nanopore data, a total of 13,164,642 reads with an N50 of 5,923 bp comprising 37 GB of data remained. The polished MaSuRCA genome assembly contained 13,709 contigs with an N50 of 142,420 bp with a total assembly size of 958 MB (Supplementary Figure 4b). In comparison, the polished Flye assembly contained 44,677 contigs with an N50 of 156,862 bp with a total assembly size of 617 MB. Based on the Illumina short reads, the estimated genome size of *C. bracteatus* is 834 MB. In terms of completeness, both the polished MaSuRCA and polished Flye assemblies were similar. Prior to polishing, the MaSuRCA assembly contained 1,288 out of 1,375 complete BUSCOs with 1,161 single-copy and 127 duplicated, 33 fragmented, and 54 missing BUSCOs. After polishing the assembly contained 1,291 out of 1,375 complete BUSCOs with 1,163 single-copy and 128 duplicated, with an additional 33 fragmented BUSCOs and 51 missing BUSCOs. In comparison, prior to polishing the Flye assembly contained 1,206 out of 1,375 complete BUSCOs with 1,115 single-copy and 91 duplicated, 97 fragmented, and 72 missing BUSCOs. After polishing the assembly contained 1,297 out of 1,375 complete BUSCOs, with 1,189 single-copy and 108 duplicated, with 30 fragmented, and 48 missing BUSCOs.

### Selection analyses

**FIGURE 6:**
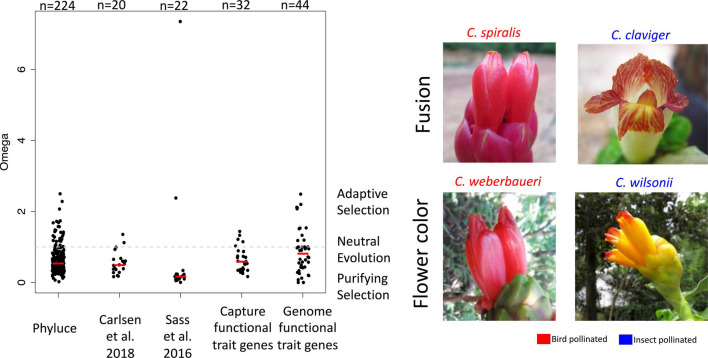
Strip plots showing the omega value for 342 loci investigated for signatures of selection in PAML, with the median value of each group of loci represented with the red line. The different columns represent different approaches of how the loci were generated, such as from Phyluce, Carlsen et al. (2018); Sass et al. (2016), and genes representing functional traits. The fifth column represents genes of functional traits isolated with consensus calling of the genomic Illumina DNA. The gray dotted line represents omega values of neutral evolution; omega values above one signifies positive selection and omega values less than one represent purifying selection. Examples of how fusion and flower color change across different pollination types are also highlighted, with genes of these function traits included in the last two columns of the strip plot.

After alignment and filtering of the 957 capture loci, 296 were used for selection analyses (Supplementary Table 6). An additional 44 candidate loci responsible for functional traits associated with pollination syndromes were used after identification in the *C. spicatus* assembled transcriptome and were extracted from the Illumina sequences using a consensus-calling approach. The breakdown of the capture loci groups were 224 loci developed with Phyluce, 20 loci from Carlsen et al. (2018), 22 loci from Sass et al. (2016), and 32 loci of functional trait genes (Figure 6). The median omega value for the loci used for the phylogeny was 0.503, while the median value for the genes underlying functional traits was slightly elevated with a value of 0.534. Not all of the phylogenetic loci were congruent in terms of selective pressure, with the Sass et al. loci having the smallest median omega value of 0.163 but also the largest omega values recovered. The Carlsen loci showed the most loci exhibiting positive selection with 30 of the 224 loci (13.4%) having omega values above one. Five of the capture genes associated with functional traits showed evidence of positive selection, which included two loci of *FLS*, two loci of anthocyanin synthase (*ANS*), and one locus of *PTL*.

Out of the additional 44 candidate genes investigated using consensus calling, 11 loci showed evidence of positive selection (omega >1), with five loci having omega values larger than two. The adaptive loci included two loci of *BIG BROTHER*, two loci of *CHS*, one locus of *CUP SHAPED COTYLEDON 2*, one locus of *DIVARICATA*, two loci of *GASA/GAST1*, one locus of *PTL*, one locus of *RADIALIS*-like, and one locus of *SUPERMAN*. Four loci, including one locus of *GASA*, two loci of *LONGIFOLIA*, and one locus of *RADIALIS*-like show a nearly infinite value of omega due to a dS value of zero or nearly zero (these four loci were not represented in Figure 6). Further investigation of these loci by comparing branch-specific omega values and the phenotypic traits scored resulted in a lack of clear patterns. Comparing tubular labellum to the branch specific omega values for *PTL* (Supplementary Figure 5a) and *SUPERMAN* (Supplementary Figure 5b) showed a slightly elevated omega value for the species with a tubular labellum (median omega = 3.62) vs. those without a tubular labellum (median omega = 3.07). The pattern of selection pressure for *PTL* is more extreme, but far from conclusive. The median omega value of species without a tubular labellum is 0.0001 (with one species, *Costus kuntzei* approaching infinity due to a very low DS value of 0.000005), while five of the species with a tubular labellum have omega values approaching infinity and four species showing evidence of purifying selection (omega <1)(Supplementary Figure 5). The patterns observed between the trait of functionally red and two of the chalcone synthase genes is quite different than for fusion. The median values of omega for *CHS4* (Supplementary Figure 6a) are identical between species that are functionally red and those that are not (0.346 vs. 0.346). In contrast, species that are not functionally red show an elevated median omega value (4.887) compared to those species that are functionally red (3.647) for *CHSY* (Supplementary Figure 6b).

### Single nucleotide polymorphism analyses

**FIGURE 7:**
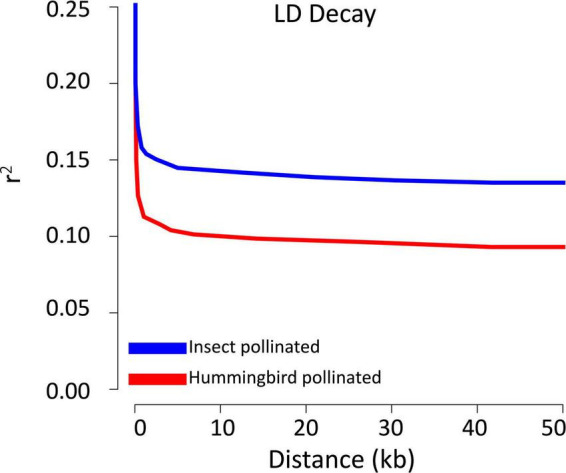
Decay of linkage disequilibrium (*r*^2^) using filtered genome wide SNPs for accessions representing either bee or bird pollination.

The initial VCF produced from GATK consisted of 115,295,456 raw SNPs. The initial filtering parameters reduced the number of SNPs to 9,992,569. Pruning for LD with an *r*^2^ value of 0.2 left 3,432,721 high quality SNPs. Calculating the LD decay by splitting the 20 samples across bee-pollinated and hummingbird-pollinated species, while excluding known hybrids, showed that the level of LD decreases rapidly and plateaus within approximately 5,000 bp. While both bee and hummingbird-pollinated species show an overall similar pattern, the two groups show differences in the baseline value (Figure 7). Bee-pollinated species show a baseline value of LD with an *r*^2^ value of 0.15, while hummingbird-pollinated species show a baseline *r*^2^ value of 0.10. In addition to differences in LD, the two different pollinator groups also display differences in heterozygosity within the variant sites. The hummingbird-pollinated species show a median heterozygosity value of 0.131, while the bee-pollinated species show a median heterozygosity value of 0.109. Notably, the two additional species that show conflicting splits in the neighbor network (see below), showed elevated levels of heterozygosity. The hummingbird-pollinated species *C. spicatus* had a heterozygosity value of 0.229, while the bee-pollinated *C. pictus* had a heterozygosity value of 0.217. If these two species are removed from the comparison, then the median heterozygosity value for the hummingbird species is 0.129 compared to 0.106 for the bee-pollinated species. The two known hybrids in the data set have a much higher median heterozygosity value of 0.288, almost double that of all other species.

The neighbornet phylogenetic network shows a similar relationship pattern for the subset of species with full genome data compared to those of the full capture data set (Supplementary Figure 7). However, there are conflicting splits across the genome data that suggest evidence of gene flow *via* hybridization. Two known hybrids in the data set, *C. vinosus* × *alenii* and *C. wilsonii* × *villosissimus* “mellow yellow,” show clear hybridization patterns with *C. villosissimus* appearing to be one of the genetic contributors to these accessions. Other species including *C. spicatus* and *Costus spiralis* (Jacq.) Roscoe which are not of known hybrid origin show conflicting splits between other species. As shown with the larger data set, the hummingbird-pollinated species do not all cluster together and neither do the bee-pollinated species. There is a larger cluster of exclusively hummingbird-pollinated species including *Costus beckii* Maas & H.Maas, *C. comosus*, *Costus productus* Gleason ex Maas, *Costus stenophyllus* Standl. & L.O. Williams, and *Costus zingiberoides* J.F.Macbr. Other hummingbird-pollinated species including *C. lasius*, *C. spiralis*, *C. spicatus*, and *C. wilsonii* each have bee-pollinated species as their closest relatives.

**FIGURE 8:**
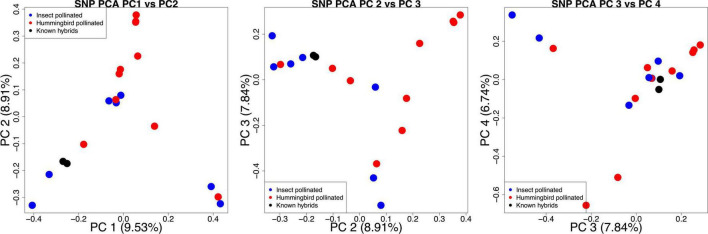
Principal component analyses using the first four principal components. Accessions are color coded if they are bee pollinated (bee), bird pollinated (red), or are known hybrids (black).

After reading in the filtered VCF containing 3,432,721 SNPs, a total of 3,104,097 biallelic SNPs were used for the PCA. The top four PC explained a total of 33.02% of the overall variation with PC1 explaining 9.53%, PC2 explaining 8.91%, PC3 explaining 7.84%, and PC4 explaining 6.74% (Figure 8). Similar to the phylogenetic network, there is no clear grouping of only bee- or hummingbird-pollinated species, with several cases where the closest relatives genetically are from different pollinator groups. The two known hybrids also cluster together, with evidence that one parent is bee pollinated (*C. villosissimus*) and the other is hummingbird pollinated (*C. wilsonii*).

## Discussion

We present here the most complete phylogeny of Neotropical *Costus* including 61 (of c.77) species with multiple exemplars capturing species-level morphologic and biogeographic diversity. Most relationships in the inferred tree are well-supported with the exception of some of the earliest branching events. Most species represented by multiple accessions are monophyletic, though the non-monophyly of some species suggests either cryptic species or the presence of multiple species complexes with ongoing gene flow. In general, trait combinations follow expectations of established ornithophilous and mellitophilous pollination syndromes. One of the more important traits for distinguishing pollination syndromes, labellum shape, appears to have evolved from a tubular to a spreading labellum multiple times followed by corresponding evolutionary shifts in other pollination-associated traits (floral color, nectar guide, lateral markings, and functional inflorescence color). Other traits, such as appendages, appear to be more labile in their evolutionary history. When single traits evolve away from expected pollination syndromes, other traits associated with the suite of traits representing established pollination syndromes appear to rapidly change to conform to a different pollination strategy and converge on the associated pollination syndrome. These rapid transition rates indicate strong adaptation to effective pollination strategies. Indeed, we see signatures of positive selection in several candidate genes associated with floral fusion and pigment production, two traits associated with the development of pollination-associated morphologies. In addition to strong morphological differences between pollination syndromes, we also see genetic differences between individuals of bee and bird pollination such as LD and levels of heterozygosity, which may be associated with outcrossing rates or geographic distance between the two parental genotypes. Even with the presence of strong morphological and genetic signatures of pollination syndromes, we do not recover evidence that the labellum shape impacts rates of diversification of Neotropical *Costus.*

We confirm the idea of a rapid radiation of the Neotropical lineage of *Costus* with short branch lengths obtained for the early nodes of the diversification of the genus in this region (Figure 1; André et al., 2016; Valderrama et al., 2020; Vargas et al., 2020). The gene tree discordance observed for the normalized quartet scores of the coalescent-based species tree topology can also be attributed to a rapid diversification, leading to incomplete lineage sorting as evidenced in many neotropical plant lineages (Schley et al., 2018; Valderrama et al., 2018; Pérez-Escobar et al., 2021; Torres Jimenez et al., 2021). Alternatively, and not mutually exclusive, the topology and the gene tree discordance could be signatures of hybridization and the resulting introgression over the history of the genus. From the phylogenetic network analysis, we observe evidence of hybridization, both in several known hybrids but also in accessions that previously have not been suspected to be hybrids (Supplementary Figure 7). However, this updated phylogeny allows testing and confirming monophyly when considering geographic and morphological variation within described species. The phylogeny also reveals close relationships of undescribed lineages, clarifying their validity as additional taxonomic units (e.g., *Costus antioquiensis* sp. nov. Maas & H.Maas., *C. oreophilus* sp. nov. Maas & Skinner, or *Costus weberbaueri* Loes.). We further confirm the need for an updated taxonomic revision that accurately reflects the diversity in the genus.

The frequency of combination of traits matching the expectations of two pollination syndromes, bee and bird pollination, as well as the strongly correlated evolution among these characters, points to the validity of the inferred relationship of those pollination syndromes with key traits, like the labellum shape (Maas, 1972; Specht et al., 2012; Maas-van de Kamer et al., 2016). Additional traits such as labellum stripes, honey mark, and inflorescence/bract coloration appear to be acquired by lineages to fine-tune the attraction to pollinators. From the clustering of SNPs genome-wide in the PCA, we do not see clusters of species that exclusively exhibit one pollination syndrome or the other (Figure 8), providing more support that lineages are evolving the necessary traits for effective pollination. The evolution of pollination related traits in *Costus* implies that if the hummingbird morphology evolved to avoid visitation by bees, as posited by the bee avoidance hypothesis (Lunau and Maier, 1995; Lunau et al., 2011), then some lineages subsequently “avoided avoiding bees” by regaining traits that attract bees, including functionally green color. The process of re-evolving traits sometimes involves the layering of structures, such as the acquisition of green appendages that obscure the presence of red bracts in *C. leucanthus* and *C. sinningiiflorus*. In this case, rather than “losing” red bracts and thereby shifting inflorescence color from red to green, leafy appendages are added creating a functionally green display. Further studies exploring the efficacy of these pigments and cost of their production in the bracts will be required to provide more insights as to the evolutionary history of bract color and the potential selective pressures acting on this inflorescence trait.

We have only limited pollination data for species of *Costus*, with pollinators observed and pollination efficacy measured for 12 species (Kay and Schemske, 2003; Bergamo et al., 2016). Observations of floral visitors of intermediate morphologies described here should be studied in the field to fully understand the selective pressure shaping the morphological variation in this radiation (Bergamo et al., 2016). Monophyletic species like *C. lima* K.Schum. with a distribution ranging from Ecuador to Costa Rica, from sea level to 2,000 m above sea level, possessing morphological variation in the indument and functional color of the inflorescence, constitute outstanding opportunities to test many hypotheses about how traits influence and are influenced by pollination biology in Neotropical plants. Questions that can be addressed include: do floral visitors and herbivory vary over the elevation gradient? How do traits associated with different pollination syndromes and defenses against herbivory actually affect floral visitors and predators along environmental gradients? Objectively measuring the spectral characteristics of the inflorescences and understanding the differences taking into account the visible spectrum of both pollinators and predators could greatly improve our understanding of the communication channels shaping these biotic interactions (Coimbra et al., 2020). Additionally exploring the signals in the UV spectrum (overlooked by our character coding) could also provide valuable information to understand the pollination biology of *Costus* (Bergamo et al., 2016). Studying the morphology of hybrids presenting trait combinations that do not match the pollination syndromes, coupled with observations of floral visitors and quantification of reproductive success, could reveal reinforcement of reproductive isolation with unfit individuals resulting from gene flow between lineages attracting different pollinators (Schley et al., 2022).

The appendages of the bracts are an interesting trait that could have a role in both the attraction of pollinators and defenses against herbivory. Appendages are marginally correlated with labellum shape and hence could play a role in pollination but are not correlated with any of the other pollination-related traits. The faster transition rates of appendages in species with a spreading labellum suggest that these structures can play a role both in the attraction of bee pollinators but also defending those inflorescences from herbivory, based on the strong correlation with the nectariferous callus that attracts ants that defend the inflorescence from predation. We hypothesize that the presence of certain types of appendages could interfere with the function of the callus or have a function protecting the inflorescences from herbivory. Also, the presence of appendages is correlated with the presence of an indument in the inflorescence, perhaps jointly playing a role against herbivory; the gain or loss of appendages mainly involves lineages with an indument already present on their inflorescences. The combination of traits displayed in *Costus* inflorescences seems to be shaped by the interactions with pollinators and predators forming a complex ecological network (Irwin et al., 2004; Dellinger, 2020). Furthermore, these traits could also play a role in attracting seed dispersers, adding another set of organismal interactions that could potentially modulate selective pressures over inflorescence traits. The complexity and interaction of many traits involved in pollination and defenses against herbivory suggest that the genetic mechanisms underlying these combinations of traits involve multiple genes and differential selection paradigms.

Convergent shifts in floral features resulting in observed shifts in pollinators are prevalent in the literature, including changes in corolla shape in Iochrominae (Solanaceae; Smith and Kriebel, 2018) and changes in stamen and stigma shape in *Salvia* L. (Lamiaceae; Kriebel et al., 2020, 2021). While many studies have investigated discrete shifts in flower color in relation to pollinators (Smith et al., 2008; McCarthy et al., 2015; Roguz et al., 2020; Garcia et al., 2021), evidence of significant pollinator-mediated selection on continuously varying flower color is lacking (Trunschke et al., 2021). Fewer studies have looked at the molecular evolution of genes responsible for pigment production, including anthocyanins (Lu and Rausher, 2003; Ho and Smith, 2016; Wheeler et al., 2021) or carotenoids (Livingstone and Anderson, 2009; Clotault et al., 2012; Cao et al., 2021). Ho and Smith (2016) found evidence of purifying selection on *CHI, F3H*, and *DFR*, providing additional evidence that gene expression may be more important than structural changes in the evolution of floral color, at least in the realm of losses of pigmentation. Across our sampling, we see evidence of positive selection on several anthocyanin producing genes including *FLS*, *ANS*, and *CHS* (Supplementary Table 6). Most shifts in flower color involve transitions from red and to green bracts and functional coloration, with a lack of transitions involving a loss of pigmentation (Figure 3). Even with the presence of flower color shifts between pollination syndromes, some anthocyanin genes show purifying selection while others in the pathway show positive selection across the phylogeny.

Our diversification analysis indicated no significant association between labellum shape and diversification (Supplementary Table 5) with the CID4 model of HiSSE showing the smallest AIC value. On one hand the lack of association is somewhat surprising since other studies have shown a strong association with diversification and traits important for pollination such as with floral pigments (Smith et al., 2010; Smith and Goldberg, 2015; Landis et al., 2018) and pollination trapping (Xue et al., 2020), corolla length (Moein et al., 2021), and corolla shape (Fernandez-Mazuecos et al., 2013). However, other traits important for pollination appear to be decoupled with diversification such as the lever mechanism in *Salvia* (Moein et al., 2021). Pollinator-mediated selection likely acts on more than one trait at a time to impact diversification, as observed by O’Meara et al. (2016), who found that the presence of a corolla, bilateral symmetry, and reduced stamen number synergistically act as key innovations to double diversification rates. The lack of impact of labellum shape on diversification may be due to the scope of the phylogeny, which was primarily focused on Neotropical *Costus*, and the rapid radiation of the group. Similar issues were previously seen in the study of nectar spurs in the Pedilanthus clade of *Euphorbia* (Cacho et al., 2010). If the phylogeny was expanded beyond the Neotropical Costaceae, the role of the labellum (and specifically labellum shape) might be considered a key innovation at the level of the family or even the order Zingiberales (Specht et al., 2012), similar to the labellum and pollinia in Orchidaceae (Zhang et al., 2017) and nectar spurs (Hodges and Arnold, 1995). However, formal testing of whether the labellum is a key innovation in Costaceae is still required.

From the selection analyses, we see that loci used for phylogenetic inference from different pipelines show different levels of selection (Figure 6). While neutral evolution of these markers might be advantageous for phylogenetic inference, many show signatures of purifying selection, lowering the overall rate of evolution (Edwards, 2009). In most cases this should not lead to biased results, though issues can arise when investigating rapid radiations due to a lack of phylogenetic signal (Morrison et al., 2004; Grummer et al., 2018; Thomas et al., 2021). Additionally, many captured genes that regulate functional traits associated with floral phenotypes show evidence of non-neutral evolution including genes associated with floral fusion and the anthocyanin pathway. Genes evolving under positive selection do not predict congruence with other markers and therefore do not necessitate exclusion from phylogenetic inference (Roje, 2014). In fact, a phylotranscriptomic study of wild tomatoes (*Solanum* sect. *Lycopersicon*) showed that 3.8% of loci showed evidence of positive selection (Pease et al., 2016). From the data presented here, we see 13.3% (39 out of 293) of the loci used for phylogenetic inference showing positive selection (Supplementary Table 6). Notably, only 293 out of 957 loci that were used for phylogenetic inference were used for PAML analyses; specifically the loci remaining after our stringent filtering parameters. Therefore, the overall proportion of loci showing positive selection is likely much lower. Moving beyond a targeted capture approach to a fully genome wide data set may be necessary to fully resolve the evolutionary history of *Costus* and the Zingiberales overall. In fact, the genomic resources for the order are still quite limited, with one chromosome scale genome assembly in *Zingiber* Mill. (Li et al., 2021), several genomes for *Musa* L. (D’Hont et al., 2012; Droc et al., 2013; Wang et al., 2019), and three draft genomes of *Costus* (Grunder, 2021).

## Conclusion

In summary we found that the recent radiation of Neotropical *Costus* encompasses unacknowledged diversity, and that the evolution of floral traits across the genus illustrates that several morphological traits associated with pollination syndromes and herbivory are correlated and are under selective pressure indicative of responses to involvement in complex ecological networks. Furthermore, selection detected in genomic regions provides evidence of the genetic underpinnings of these pollination-associated traits. The evidence presented here will guide many hypotheses, most likely to be tested at the population or species-pairs level, addressing the genomic mechanisms and the evolutionary ecology of these traits associated with the morphological diversity and evolution of pollination syndromes across this diverse lineage of the Neotropical Costaceae.

## Data availability statement

The datasets and scripts generated for this study can be found in the Open Science Framework https://osf.io/fkj2x and raw reads in NCBI BioProject http://www.ncbi.nlm.nih.gov/bioproject/639561. Raw sequencing data for whole genome sequencing and transcriptome sequencing, including Illumina and Nanopore data, are available with the following SRA numbers: SRR18516534 and SRR18516558 (numbers for specific accessions can be found in Supplementary Table 3). Both versions of the assembled *Costus bracteatus* genome are available on CoGe (https://genomevolution.org/coge/) with genome IDs 63651 (polished Flye assembly) and 63654 (polished MaSuRCA assembly).

## Author contributions

CDS and AA conceived of the project and gathered the preliminary data. PM, HM-K, TA, and DS provided cultivated and field-collected materials of otherwise impossible-to-get taxa representing documented morphologic and biogeographic variation. EV and JL collected and analyzed the data and wrote the manuscript. CDS, DS, PM, HM-K, TA, and EV contributed to tissue collection, sampling, and database management. MP-V, AA, HP, NG, and CG collected the data and contributed to database management. CS collected and analyzed phylogenetic data. All authors contributed to the article and approved the submitted version.

## Footnotes

1
https://osf.io/fkj2x

2
https://github.com/FelixKrueger/TrimGalore

3
sourceforge.net/projects/bbmap/

4 Maas, P. J. M., Maas-van de Kamer, H., Andre, T., Skinner, D., Valderrama, E., and Specht, C. D. (Submitted). A revision of the neotropical Costaceae Nakai: a sixty-year struggle. Part 1: new species. *Phytokeys.*

5
https://github.com/rrwick/Porechop

6
https://github.com/acorg/dark-matter

## Supplementary material

Supplementary Figure 1 Maximum likelihood (IQtree) concatenation phylogeny of 945 loci. The phylogeny is rooted with African *Costus* and Neotropical Costaceae accessions. Samples with wg in the ID indicates that the accession was included by assembling the target loci from whole genome sequencing data.

Supplementary Figure 2 Topologies obtained with concatenation vs. coalescent-based species tree analyses, showing just one node difference between the two. Branch lengths are proportional to the number of substitutions for the IQtree result and to coalescent units in the ASTRAL result.

Supplementary Figure 3 Species tree reconstruction by ASTRAL with local posterior probabilities above the branches. Pie charts illustrate the quartet scores for each node for the 945 loci, with red representing the current topology, blue the second most favored topology, and white the remaining one. Equal branch lengths were used to allow the reader to distinguish support values.

Supplementary Figure 4 Assembly stats for the **(a)** *Costus spicatus* transcriptome assembly and **(b)** genome assembly of *Costus bracteatus*. The N50 of each assembly is denoted with a blue line. Number of BUSCO genes from the embryophyta library (1,375 total genes) is shown for complete and single copy genes, complete and duplicate genes, fragmented, and missing genes.

Supplementary Figure 5 Branch specific omega values for **(a)** *PETAL LOSS* (*PTL*) and **(b)** *SUPERMAN* across the gene tree of 20 accessions with whole genome Illumina sequencing. Omega values representing purifying selection (<1) are in blue, values close to neutral evolution (∼1) are in purple, and values of positive selection (>1) are shades of red. Ornithophilous and mellitophilous syndromes are indicated with a bee or hummingbird icon.

Supplementary Figure 6 Branch specific omega values for **(a)** *CHS4* and **(b)** *CHSY* across the gene tree of 20 accessions with whole genome Illumina sequencing. Omega values representing purifying selection (<1) are in blue, values close to neutral evolution (∼1) are in purple, and values of positive selection (>1) are shades of red. Ornithophilous and mellitophilous syndromes are indicated with a bee or hummingbird icon.

Supplementary Figure 7 NeighborNetwork of the 20 species with Illumina genome sequencing highlighting conflicting splits. Accessions are coded as bee pollinated (blue), bird pollinated (red), or known hybrids (black).

Supplementary Table 1 Genus, species, identifiers, collector, number, and natural history collection for all the accessions included.

Supplementary Table 2 Binary traits scored for each accession including labellum shape (tubular/spreading), bract color (red/non-red), bract appendages (present/absent), honey mark (present/absent), labellum stripes (present/absent), functional color (red/non-red), callus (present/absent), and inflorescence indument (present/absent).

Supplementary Table 3 Genomic sequencing information for 20 accessions including the accession number, number of filtered SNPs retained, SRA number for Illumina sequencing, and SRA for Oxford Nanopore.

Supplementary Table 4 Phylogenetic D statistic estimates and *p*-values for the tests of phylogenetic D being significantly different from 0 (as expected under a Brownian threshold model) and 1 (random) for the analyzed traits. Additionally, the Chi-squared estimate and associated *p*-value of the likelihood ratio test comparing ER vs. ARD models fitted for each trait are included.

Supplementary Table 5 Log-likelihood values (lnL), Akaike Information Criterion (AIC), and corrected AIC (AICc) for the different models of diversification fitted to the phylogeny of Neotropical *Costus.* The binary state speciation and extinction model (BiSSE) as well as the hidden state speciation and extinction model (HiSSE) were fitted with the labellum shape character. The constant rate and additional hidden state models (CID2 and CID4) are independent from the labellum shape.

Supplementary Table 6 Omega values for each locus used in PAML, including which set that locus belongs to in the strip plot summary.
