# Supplementary figures and images for: The genetic mechanisms underlying the convergent evolution of pollination syndromes in the Neotropical radiation of Costus L
Source: Front Plant Sci. 2022 Sep 8;13:874322. doi: 10.3389/fpls.2022.874322 (PMC9493542; doi:10.3389/fpls.2022.874322)

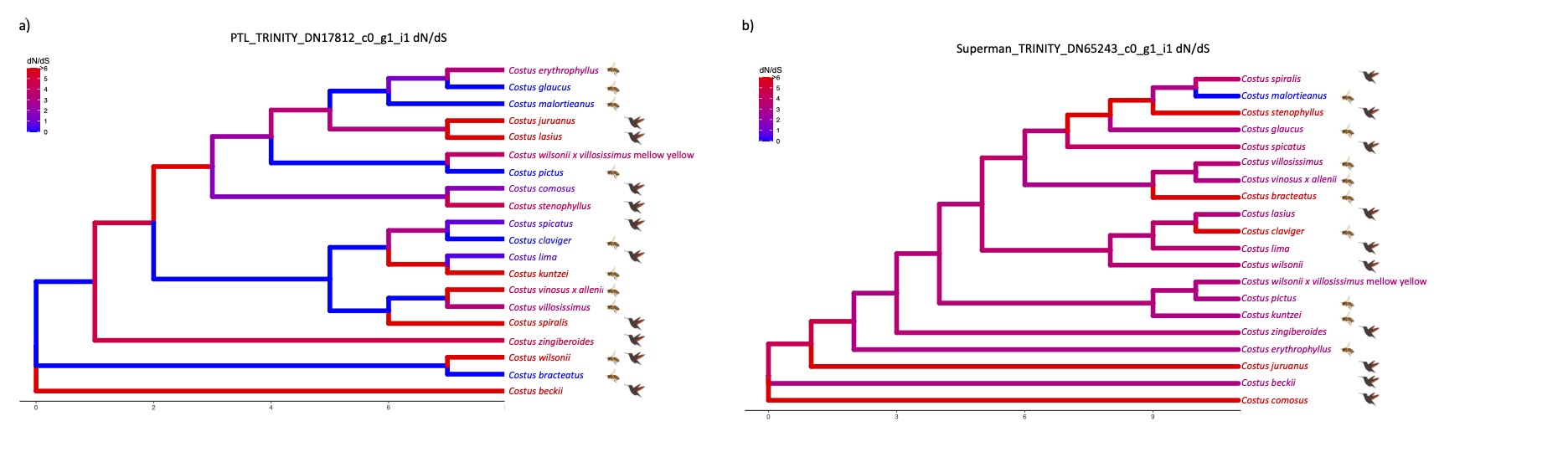

Supplement: Supplementary Figure 5 — Branch specific omega values for (a) PETAL LOSS (PTL) and (b) SUPERMAN across the gene tree of 20 accessions with whole genome Illumina sequencing. Omega values representing purifying selection (<1) are in blue, values close to neutral evolution (∼1) are in purple, and values of positive selection (>1) are shades of red. Ornithophilous and mellitophilous syndromes are indicated with a bee or hummingbird icon. [file Image_5.JPEG]

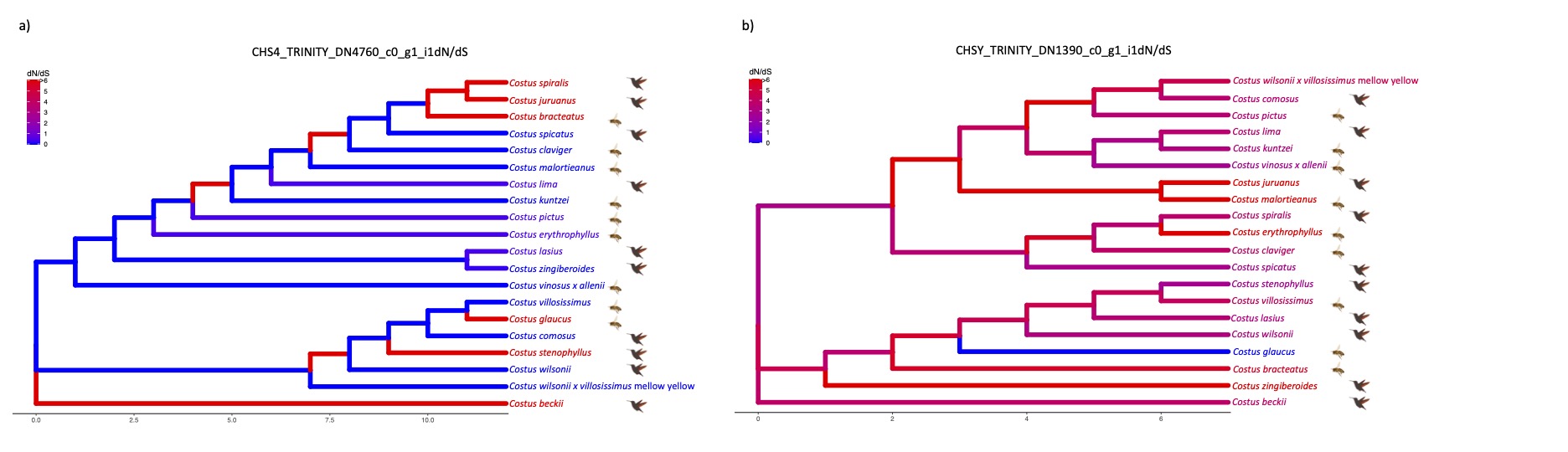

Supplement: Supplementary Figure 6 — Branch specific omega values for (a) CHS4 and (b) CHSY across the gene tree of 20 accessions with whole genome Illumina sequencing. Omega values representing purifying selection (<1) are in blue, values close to neutral evolution (∼1) are in purple, and values of positive selection (>1) are shades of red. Ornithophilous and mellitophilous syndromes are indicated with a bee or hummingbird icon. [file Image_6.JPEG]
