# Supplementary material for: The genetic mechanisms underlying the convergent evolution of pollination syndromes in the Neotropical radiation of Costus L
Source: Front Plant Sci. 2022 Sep 8;13:874322. doi: 10.3389/fpls.2022.874322 (PMC9493542; doi:10.3389/fpls.2022.874322)
Supplement: Supplementary Table 3 — Genomic sequencing information for 20 accessions including the accession number, number of filtered SNPs retained, SRA number for Illumina sequencing, and SRA for Oxford Nanopore. [file Table_3.docx]

Supplemental Table 3. Accession numbers for samples used for whole genome sequencing and transcriptome sequencing.

| Species | Accession number | Pollinator type | Number of filtered SNPs | SRA genome Illumina | SRA Nanopore |
| --- | --- | --- | --- | --- | --- |
| *Costus beckii* | R3394 | Hummingbird | 3,350,337 | SRR18516558 |  |
| *Costus bracteatus* | R3030 | Insect | 3,411,294 | SRR18516557 | SRR18516543 |
| *Costus claviger* | R3219 | Insect | 3,363,316 | SRR18516546 |  |
| *Costus comosus* | R3031 | Hummingbird | 3,362,559 | SRR18516540 |  |
| *Costus erythrophyllus* | R3026 | Insect | 3,366,647 | SRR18516539 |  |
| *Costus glaucus* | R3364 | Insect | 3,320,446 | SRR18516538 |  |
| *Costus juruanus* | R2693 | Hummingbird | 3,357.431 | SRR18516537 |  |
| *Costus kuntzei* | R3086 | Insect | 3,369,472 | SRR18516536 |  |
| *Costus lasius* | R3127 | Hummingbird | 3,367,151 | SRR18516535 |  |
| *Costus lima* | R3362 | Hummingbird | 3,319,558 | SRR18516534 |  |
| *Costus malortieanus* | R1562 | Insect |  | SRR18516556 |  |
| *Costus pictus* | R3172 | Insect | 3,385,569 | SRR18516555 |  |
| *Costus spicatus* | R3385 | Hybrid/ Hummingbird | 3,383,036 | SRR18516553 |  |
| *Costus spicatus* | Cornell | Hummingbird |  | SRR18516545, SRR18516544 | SRR18516542, SRR18516541 |
| *Costus spiralis* | R3218 | Hummingbird | 3,372,383 | SRR18516552 |  |
| *Costus stenophyllus* | R2613 | Hummingbird | 3,333,878 | SRR18516551 |  |
| *Costus villosissimus* | R3074 | Insect | 3,342,764 | SRR18516550 |  |
| *Costus vinosus x allenii* | R3353 | Hybrid/Insect | 3,421,019 | SRR18516549 |  |
| *Costus wilsonii* | R3092 | Hummingbird | 3,358,790 | SRR18516548 |  |
| *Costus wilsonii x villosissimus*  *sp mellow yellow* | R3091 | Hybrid | 3,424,116 | SRR18516554 |  |
| *Costus zingiberoides* | R3406 | Hummingbird | 3,366,113 | SRR18516547 |  |
